# Molecular Mechanisms of Chemoresistance Induced by Cisplatin in NSCLC Cancer Therapy

**DOI:** 10.3390/ijms22168885

**Published:** 2021-08-18

**Authors:** Jolanta Kryczka, Jakub Kryczka, Karolina H. Czarnecka-Chrebelska, Ewa Brzeziańska-Lasota

**Affiliations:** 1 Department of Biomedicine and Genetics, Medical University of Lodz, 92-213 Lodz, Poland; karolina.czarnecka@umed.lodz.pl (K.H.C.-C.); ewa.brzezianska@umed.lodz.pl (E.B.-L.); 2 Institute of Medical Biology, Polish Academy of Sciences, 93-232 Lodz, Poland; jkryczka@cbm.pan.pl

**Keywords:** non-small cell lung cancer, cisplatin, chemoresistance molecular mechanisms, tumour microenvironment, DNA repair mechanisms

## Abstract

Cancer cells utilise several mechanisms to increase their survival and progression as well as their resistance to anticancer therapy: deregulation of growth regulatory pathways by acquiring grow factor independence, immune system suppression, reducing the expression of antigens activating T lymphocyte cells (mimicry), induction of anti-apoptotic signals to counter the action of drugs, activation of several DNA repair mechanisms and driving the active efflux of drugs from the cell cytoplasm, and epigenetic regulation by microRNAs (miRNAs). Because it is commonly diagnosed late, lung cancer remains a major malignancy with a low five-year survival rate; when diagnosed, the cancer is often highly advanced, and the cancer cells may have acquired drug resistance. This review summarises the main mechanisms involved in cisplatin resistance and interactions between cisplatin-resistant cancer cells and the tumour microenvironment. It also analyses changes in the gene expression profile of cisplatin sensitive vs. cisplatin-resistant non-small cell lung cancer (NSCLC) cellular model using the GSE108214 Gene Expression Omnibus database. It describes a protein-protein interaction network that indicates highly dysregulated TP53, MDM2, and CDKN1A genes as they encode the top networking proteins that may be involved in cisplatin tolerance, these all being upregulated in cisplatin-resistant cells. Furthermore, it illustrates the multifactorial nature of cisplatin resistance by examining the diversity of dysregulated pathways present in cisplatin-resistant NSCLC cells based on KEGG pathway analysis.

## 1. Lung Cancer from a Global Perspective

Globally, lung cancer continues to be the primary cause of cancer deaths in both men and women, being the most common cancer type for men, constituting 22% of total cancer incidence, and the third most common in women, in whom it represents 8.4% total cancer incidence after breast and colorectal cancers [1,2,3]. About 2.1 million new lung cancer cases were diagnosed worldwide in 2018, which accounts for 11.6% of the world’s total cancer incidence. Overall, lung cancer mortality amounted to 1.8 million in 2018, accounting for 18.4% of cancer deaths [3]. The five-year survival index for early-stage lung cancers exceeds 50% [4]. This high mortality is primarily because only 15% of these cancers are discovered in the early stages; therefore, despite the presence of advanced modalities for treatment, most cancers are diagnosed at an advanced stage, and the overall five-year survival rate is only about 15% [4,5].

### 1.1. Histopathological Type

Lung cancer may be classified into two major groups: small cell lung cancer (SCLC) and non-small cell lung cancer (NSCLC) according to histopathological diagnosis [6,7]. NSCLC accounts for approximately 80–85% of all lung cancer cases [2,8,9]. It comprises two predominant histological subtypes: adenocarcinoma (ADC, approximately 40–50% cases) squamous cell carcinoma (SCC, approximately 20–30% cases) [6,8,10].

### 1.2. Treatment of NSCLC

The choice of treatment of NSCLC depends on the histological subtype and genetic subtype of the tumour and disease stage, comorbidity, and performance status [11]. In cases of early-stage NSCLC with no contraindications, surgical resection of the tumour is indicated; while unresectable tumours can be controlled to a certain degree with radiation therapy, only a small number of patients demonstrate positive outcomes [4,12]. Alternatively, patients with locally advanced unresectable lung cancer may achieve long-term survival by treatment with a combination of radiation therapy and chemotherapy [12]. One of the “first choice” drugs used to treat various solid tumours, including lung cancer, is cisplatin, discovered in 1965 and approved by the Food and Drug Administration in 1978 [13]. Furthermore, in the case of the advanced metastatic form of lung cancer, improved survival and palliation of symptoms may be achieved with chemotherapy, targeted agents, and other supportive measures [12].

### 1.3. The Effect of Cisplatin

NSCLC patients are less sensitive to chemotherapy based on the doublet of cisplatin, cis-diamminedichloroplatinum (II), versus SCC patients [11,12]. These compounds have a well-known mechanism of action [14]. Cisplatin generally enters cells by passive diffusion, where it is then activated [15,16]. In the cytosol, its chloride ligands are replaced by water molecules, generating positively charged mono- and bi-aquated forms of cisplatin that react with various membrane and cytoplasmic components, as well as nuclear DNA and RNA [14,17]. 

Aquated species of cisplatin can form covalent bonds with endogenous nucleophilic such as methionine, cysteine-containing peptides, and polypeptides, including reduced glutathione (GSH) and metallothioneins (MT) [17,18]. These interactions increase oxidative stress via depletion cell of reducing equivalents, resulting in cytotoxic effects; however, those molecules additionally function as a cytoprotective buffer, as chemically active cisplatin is inactivated by reacting with them, thus protecting more vital targets (DNA) [17,19]. 

Cisplatin also causes the formation of intrastrand and interstrand cross-links in DNA [15,20]. Cross-links between guanine bases are induced by cisplatin, carboplatin, and oxaliplatin [20,21]. While cisplatin and carboplatin form identical cross-links, those formed by oxaliplatin include the bulky 1,2-diaminocyclohexane group in the adduct [22]. Only minor DNA damage is needed to disrupt replication and transcription [23]. However, it may be an oversimplification that the cytotoxic properties of cisplatin are based on its binding to nuclear DNA, mainly via intrastrand DNA cross-links, leading to cell cycle arrest and subsequent apoptosis [16]. 

## 2. Mechanisms Underlying Cisplatin Resistance

**Figure 1:**
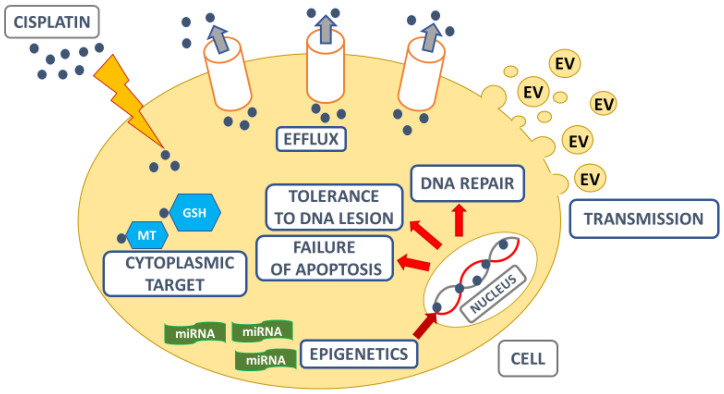
Molecular mechanisms of cisplatin resistance. GSH, reduced glutathione; MT, metallothioneins; EV, extracellular vesicles.

Compounds based on cisplatin are used in the advanced disease of NSCLC treatment and adjuvant chemotherapy [20]. However, this treatment entails a multipronged adaptive response in malignant cells, which renders them less susceptible to the antiproliferative and cytotoxic effects of the drugs, resulting in the resumption of proliferation [16,20,21]. These mechanisms allow the cancer cell to survive and progress in human organisms, thus develop resistance to therapy [24]. Such resistance is a significant cause for therapeutic failure of NSCLC, leading to tumour recurrence and disease progression [25]. The mechanisms underlying cisplatin resistance are multifactorial [16]. A significant role is played by tolerance or repair of cisplatin-DNA adducts. In addition, resistance has been associated with the induction of anti-apoptotic signals, the active efflux of drugs from the cell cytoplasm, epigenetic regulation by miRNA, deregulation of growth regulatory pathways by acquiring growth factor independence, suppression of the immune system, and low expression of antigens that activate T lymphocyte cells (mimicry) [16,24,26,27]. All these mechanisms appear to play crucial roles in cisplatin resistance. Broader knowledge of the extensive interactions of cisplatin taking place in the cytoplasm and nucleus and the multifactorial nature of resistance will enable a complete understanding of cisplatin resistance in patients with NSCLC (Figure 1) [16].

### 2.1. Repair of DNA Damage

Platinum compounds are believed to be the most active anticancer agents currently used in clinical therapies of NSCLC. Their cytotoxic activity is based on their ability to form DNA adducts [28,29]. Cisplatin induces both intrastrand cross-links, comprising around 90% of cases, and interstrand cross-links (ICLs), comprising 5–8% of DNA adducts. Generally, there are two forms of intrastrand cross-link: the 1,2-intrastrand cross-link between two adjacent purines, being the predominant form, and the 1,3-intrastrand adducts [28,29,30]. ICLs link two bases on the opposite strands of DNA [30].

The formation of platinum adducts is particularly deleterious, as a distortion of the DNA double helix blocks DNA replication and transcription. In addition, if the damage is not repaired, they can lead to single-strand breaks (SSBs), double-strand breaks (DSBs), and chromosomal rearrangements. Upregulation of processes such as DNA damage response (DDR) and DNA damage tolerance (DDT) is advantageous to cancer cells due to allowing them to resist these damaging lesions. For example, many types of cancers exhibiting chemoresistance, including lung cancer, demonstrate upregulated DDR and DDT pathways [30]. In NSCLC, many processes aimed to remove or repair the DNA lesions activate the cellular DDR [30,31]. Depending on the type and location of DNA damage, several repair pathways exist, such as nucleotide excision repair (NER), homologous recombination repair (HRR), nonhomologous end joining (NHEJ), and translesion synthesis (TLS) or post-replication repair (PRR) [28,30,32,33]. These repair mechanisms demonstrate different degrees of specificity and fidelity; however, they may be mutually complementary in certain types of damage [30,33]. If the DNA lesions are not repaired before replication, the damaged DNA cannot be utilised as a template for replication by high fidelity DNA polymerases. Damage results in the replication fork stalling and the development of a replication gap. To complete DNA replication across the lesion and, consequently, enable cell survival, cells utilise error-free or error-prone lesion bypass mechanisms to synthesise DNA. The template must be switched from the damaged to undamaged DNA strand to ensure error-free lesion bypass and allow synthesis past the lesion [30]. 

The mechanism of error-prone repair involves a concerted and coordinated interplay between different cell-cycle checkpoints and DDT pathways. The primary pathways are homologous recombination (HR), homologous recombination repair, Fanconi anaemia (FA), nucleotide excision repair, and translesion synthesis (TLS) [28]. ICL repair begins by TLS using low fidelity DNA polymerases, preparing the leading template strand for repair by the HR pathway. Stalled replication forks activate the FA pathway, which detects and repairs the stalled replication forks with the common biochemical FA/BRCA HRR pathway [30]. 

#### 2.1.1. Nucleotide Excision Repair

Intra-strand DNA cross-links are relatively straightforward. Only one strand is damaged, and the second strand remains available as a template for repair synthesis. These adducts are most commonly repaired by NER [28,32]. NER involves several proteins used for damage recognition and damage excision, as well as a helicase [34]. Lesions in the DNA helix are recognised by the XPC-RAD23B damage recognition protein complex, which binds to the DNA strand [34]. There are 24–32 nucleotides in length. An oligonucleotide is excised on both sides of the lesion on the DNA strand, and the resulting gap is patched by repair synthesis and ligation [30,32,34].

Additionally, NER acts as an essential mediator of responsiveness to cisplatin-based chemotherapy. Recent studies showed that the lung cancer cell line, Calu-1, which is moderately resistant to cisplatin, exhibited an elevated level of NER factors, participating in DNA repair including XPA, XPC-hHR23B, XPG, ERCC1-XPF, TFIIH, PCNA, and DNA ligase [33].

#### 2.1.2. Post-Replication Repair

Studies on Saccharomyces cerevisiae yeast have provided a good understanding of the activity of PRR pathways [35]. Stalled DNA replication is typically restarted by PRR pathways such as TLS or template switching (TS). Both pathways are regulated by ubiquitination of the proliferating cell nuclear antigen (PCNA) at Lysine 164 (K164) or Lysine 63 (K63) [36].

The TLS pathway is initiated by a protein complex formed by RAD6 (an E2 ubiquitin-conjugating enzyme) and RAD18 (an E3 ubiquitin ligase) [30,36]. The RAD6-RAD18 complex (an E2–E3 complex) induces posttranslational monoubiquitination of PCNA at K164 (monoUb-PCNA); such monoubiquitination is the primary modification of PCNA in mammals. Following this, monoUb-PCNA recruits one of the four Y-family specialised polymerases TLS: Pol κ, Pol η, Pol τ, or Rev1 [30]. Interactions between the ubiquitin (Ub) moiety of monoUb-PCNA and the Ub-binding domains allows the TLS polymerases to bind to the stalled 3′-ends or to the damage sites, thus allowing replication over the DNA lesion [30,36]. Following the incorporation of the nucleotide opposite the damage site, the insertion of TLS polymerase is replaced by polymerase Pol ζ, an error-prone polymerase belonging to the B-family formed as a heterodimeric complex of Rec3/Rev7 [30]. In PCNA-dependent TLS, Pol ζ forms a complex with the Pol31 and Pol32 subunits of Pol β (Rev3-Rev7-Pol31-Pol32, referred to as Pol ζ4) [30,37]. Pol ζ contains active sites that accommodate distorted DNA bases and base-pair mismatches and extends the TLS patch by ~18 nucleotides [30]. This extension step allows the lesion to escape detection because TLS polymerases do not have intrinsic exonuclease activity. Incorporating faulty nucleotides by low-fidelity TLS polymerases may increase spontaneous mutagenesis, resulting in platinum-chemotherapy tolerance and toxicity within normal cells [30,36,38]. When the strand is extended past the DNA lesion, Pol ζ is replayed by the high-fidelity DNA polymerase [30]. 

The TS pathway is promoted by additional factors, such as MMS2-UBC13 (a UEV–E2 complex) and HLTF (an E3 ligase), which are functional homologues of yeast Rad5 [30,36,39]. This stable complex allows polyubiquitination of PCNA (polyUb-PCNA); however, PCNA polyubiquitination occurring in response to alkylating agents is ~20-fold slower than monoubiquitination. PCNA polyubiquitination predominantly occurs via en bloc transfer of preformed ubiquitin chains, initiated by the MMS2–UBC13 complex, which initiates the formation of ubiquitin chains at the K63 linkages of PCNA. Briefly, HTLF forms a thiol-linked ubiquitin chain on UBC13, which is then transferred to RAD6~ubiquitin to form RAD6~ubiquitinn+1; subsequently, RAD18 transfers the resultant Ub chain to PCNA en bloc. In the TS pathway, PolyUb-PCNA stimulates the release of the stalled primer end from the damaged template, which then joins with the newly synthesised daughter strand of the sister chromosome. The TS pathway is essentially error free, as the repair is based on an undamaged template [30,36].

Recently studies suggested that PRR pathways contribute to the chemoresistant phenotype in NSCLC. The TLS function of Pol ζ is believed to play a crucial role in its ability to enhance resistance to platinum-based chemotherapies. Doles et al. showed that reducing the Pol ζ activity can make an intractable lung cancer model of NSCLC susceptible to cisplatin-based chemotherapy. Inhibition of Rev3L expression or activity may be particularly effective, as cisplatin treatment increases Rev3L mRNA levels, and elevated Rev3L was shown to promote cisplatin resistance [40]. Additionally, Ceppi et al. confirmed the association of platinum sensitivity with the endogenous Pol η mRNA levels in several NSCLC cell lines. Their results indicate a linear relationship between basal Pol η levels and in vitro cisplatin sensitivity. Endogenous Pol η mRNA presented a significantly higher level in the most cisplatin-sensitive NSCLC cell lines, while the lower level was observed in resistant cell lines (with a comparable degree of cisplatin resistance) [41]. 

#### 2.1.3. Fanconi Anaemia and ICL Repair

FA is an autosomal recessive genetic disease that is caused by mutations in the Fanconi anaemia protein cluster. It is characterised by hypersensitivity to various agents that induce ICL and chromosomal instability and can favour the development of various cancers [30]. FA pathways function mainly during the S phase and are involved in ICL repair [30,42]. Additionally, FA or FA-like proteins have been found to mediate cellular resistance NSCLC against agents which induce ICL [33,42].

In response to ICL, the FA pathway induces phosphorylation of FANCI by the FA core complex, which contains MHF1-2, FAAP24, and large multi-subunit ubiquitin E3 ligase [30,42]. The checkpoint kinase ATR phosphorylates FANCD2 at threonine 691 (T 691) and at serine 717 (S 717) [30,42,43]. Such phosphorylation of FANCD2 induces enhanced cellular resistance to ICL stimulating agents and is also required to establish the intra-S-phase checkpoint response [42]. This modification helps stabilise the replication forks but is not required for FA pathway activation [30]. 

MHF1-2 and FAAP24 recruit FANCL, a large multi-subunit ubiquitin E3 ligase (FA core complex). FANCL contains a plant homeodomain (PHD) that catalyses the mono-ubiquitylation of FANCD2-FANCI [42]. The complex of FANCL with UBE2T and UBE2W (enzymes E2) induces monoubiquitination of FANCD2 at Lysine 561 (K561); additionally, FANCL in complex with UBE2T promote monoubiquitination of FANCI at Lysine 521 (K521). FANCD2 monoubiquitination is an essential modification for the FA network and is also considered a surrogate marker of activation [30]. 

Protein ubiquitylation regulates various biological processes, including DNA damage checkpoints and DNA repair pathways. The monoubiquitination of FANCD2 and FANCI results in the FANCI/FANCD2 complex creation, which is translocated and assembled into DNA repair sites. The complex also recruits FAN1 endonucleases that colocalise FA proteins (PALB2, BRCA2, FANCJ, RAD51C, and SLX4) to remove the ICL through NER [30,42]. It is believed that TLS polymerases are then recruited to repair the damage—RAD6/RAD18 promotes the polymerases following induction by monoUb-PCNA. However, the exact sequences of repair pathways concerning NER/TLS remain unclear. Probably FANCI/FANCD2 complex creates the incision at the site of the ICL, and then TLS fills the gap at the lesion. Alternatively, TLS may promote the incision at the site of DNA damage, and the FANCI/FANCD2 complex then induces TLS activity nearby [30].

Given that the FA pathway plays an essential role in response to therapy-induced DNA interstrand cross-links, cancers with defective FA pathways are probably more sensitive to cisplatin-based therapy [44]. Ping et al. found that the cisplatin-resistant NSCLC cell line A549/DR exhibits a significantly elevated expression level of the FA factors compared to its parent cell line A549 [33]. Additionally, previous studies that used specific small molecule inhibitors or RNA targeting FA pathway-associated genes showed a variable sensitisation of tumour cells to cisplatin [33,44,45,46].

#### 2.1.4. Homologous Recombination Repair

In NSCLC, in response to DSBs, the HRR pathway is activated during the S and G2 phases of the cell cycle [30,33,47]. HRR uses the undamaged sister chromatid as a template. The procedure consists of three main steps: end resection, strand invasion, and resolution [30]. In the initial step involving nucleolytic resection, MRE11-RAD50-NBS1 (MRN) complex and the 5′ to 3′ exonuclease Exo1 are activated to resect nucleotides and extend the annealed 3′-single-stranded DNA (3′-ssDNA) overhangs; MRN and Exo1 are activated singly or in combination with Bloom’s syndrome RecQ helicase-like protein (BLM) and helicase/endonuclease DNA2 [30,48,49]. Following activation, the 3′-ssDNA tails generated via DNA end resection are stabilised by replication protein A (RPA) [30,49]. In a process aided by the mediator proteins Rad52 or BRCA2 with localiser PALB2, RPA is removed and exchanged for the Rad51 recombinase. These proteins are involved in Rad51-ssDNA filament formation and protect Rad51 from removal [30,48,49]. The Rad51-ssDNA complex is a right-handed helical polymer, with the DNA being held in an extended conformation [49]. The complex performs a “presynaptic” search for a homologous sequence in double-stranded DNA, leading to the production of heteroduplex DNA (hDNA) and the formation of a transient structure known as the displacement loop or D-loop structure [30,48,49,50]. During homologous paring, the activity of Rad51 is stimulated by Rad 54, a member of the Swi2/Snf2 family of chromatin remodelling proteins/ATPases [48]. 

When the 3′ overhang of the invading strand is free from RAD51, it serves as a primer for initiation of DNA synthesis, thus allowing the extension of the D-loop structure [30,48,49]. It is currently unclear which replication machinery is used for this elongation, but it has been shown that Pol η of TLS polymerases particularly demonstrates an affinity for D-loop elongation [30]. 

After elongation, two pathways can be utilised to resolve the D-loop: the DSB repair (DSBR) and synthesis-dependent strand annealing (SDSA) models [30,49,50]. In the SDSA pathway, D-loop extension continues for a short distance. The D-loop is disassembled by dissociating the newly synthesised strand with the ssDNA associated with the other DSB end; this step is performed by the regulator of telomere elongation helicase 1 (RTEL1) [30,49,50]. This pathway is preferred for mitotically dividing cells, and it always provides a non-crossover gene conversion product [50]. In contrast, in the DSBR pathway, the gap is filled by capturing and ligating the second end to create a double Holliday Junction (dHJ) [30,50]. This mechanism involves BLM helicase, an ATP-dependent 3′-5′ DNA helicase used to unwind D-loops [30]. Resolution of the dHJ can lead to the formation of a crossover or non-crossover product [50].

Emerging data on the role of HR in the repair of cisplatin adducts is becoming increasingly significant and highlights the importance of this DNA repair process in NSCLC chemoresistance. Previous studies revealed a variable sensitisation of tumour cells to cisplatin depending on the activity of HR pathway-associated genes [47]. Moreover, in another study, Ping et al. found that the cisplatin-resistant NSCLC-derived cell line A549/DR exhibits dramatically elevated expression levels of the HR factors compared to both its parent cell line A549 and its moderately resistant to cisplatin Calu-1 cell line. Additionally, they proved that the depletion of HR associated factors correlates with the increased ICL damage and decreased HR repair, thus leading to the NSCLC hyper sensitisation to cisplatin [33].

### 2.2. Apoptosis

The major goal of cancer chemotherapy is to force tumour cells to execute apoptosis following exposure to anticancer agents, such as cisplatin used in the treatment of NSCLC. However, cellular damage caused by chemotherapeutics must pass a certain threshold level to trigger programmed cell death [51]. The effector phase of apoptosis involves several pro- and anti-apoptotic proteins, including pro-apoptotic Bcl-2-associated X protein (Bax), Bcl-2 homologous antagonist/killer (Bak), BCL2 associated agonist of cell death (Bad) and anti-apoptotic B-cell lymphoma 2 (Bcl-2), B-cell lymphoma-extra-large (Bcl-XL), and Bcl-2-like protein 2 (Bcl-w). However, cancer cells commonly demonstrate mutations in the genes involved in various signalling pathways, including apoptotic ones, thus often leading to their dysfunction. This in effect may result in the formation of resistance to cisplatin, as any interference that mediates the induction of anti-apoptotic signal transduction or inhibition of pro-apoptotic pathways, including transcriptional and translational responses, is a potential mechanism of drug resistance.

Furthermore, apoptosis induced by cisplatin in both cisplatin-sensitive and cisplatin-resistant cancer cells leads to increased Bax mRNA and Bak protein levels and decreased expression of Bcl-2. While cisplatin activates a robust apoptotic response based on activation of the JNK pathway in cisplatin-sensitive cancer cells, no such response is observed in resistant cells [16,52]. Additionally, three essential mediators of chemoresistance in cancer cells are X-linked inhibitors of apoptosis protein (Xiap), Akt, and p53 [51]. Of the three, p53 is the primary tumour suppressor. Cisplatin treatment of cancer cells leads to p53 activation and its stabilisation by phosphorylation at the Ser15 and/or Ser20 sites, which inhibit the p53 association with E3 ubiquitin ligase mouse double minute 2 (Mdm2). Finally, this blocks the degradation of p53, which in normal cells is regulated by Mdm2. In contrast, the cell survival factor Akt inhibits apoptosis. Akt participates directly in suppressing pro-apoptotic proteins and indirectly induces growth factor-mediated and cytokine-mediated expression of anti-apoptotic protein [16,52]. 

Additionally, 34% of patients with NSCLC have a mutation of the tumour suppressor gene TP53 that encodes p53 protein (including nonsense mutation and pro-oncogenic “gain-of-function mutation”), which has been associated with frequent smoking [52]. Such mutations in TP53, including “gain of function mutation”, cause the dysregulation of multiple signalling cascades, such as apoptotic pathways [52,53]. For example, p53 status strongly influences the action of cyclin-dependent kinase inhibitor 1A (CDKN1A), which regulates G1/S and G2/M checkpoints and is transiently recruited to facilitate cisplatin-induced DNA damage. Upregulation of CDKN1A allows cells to acquire a highly aggressive phenotype and to escape cell cycle blockage and apoptosis [54]. Cisplatin also accumulates in mitochondria, forming adducts with mitochondrial DNA. This process leads to the impaired synthesis of proteins involved in the electron transport chain and an increase of the intracellular ROS level [52]. 

p53 also demonstrates an antioxidant function by regulating a wide range of antioxidant genes. Furthermore, ROS impair the function of tumour suppressors such as p53 by inflicting DNA damage. They also activate the PI3K/Akt pathway involved in cell survival and proliferation by epidermal growth factor receptor (EGFR), thus further enhancing resistance to chemotherapy among cancer cells. Moreover, Akt is involved in the activation of EGFR and down-regulation of ROS. The PI3K/Akt pathway inhibits ROS production by regulating the expression of Forkhead Box Protein O1 (Foxo1) transcription factor and Caspase-3, which are involved in the intrinsic apoptosis. Thus, EGFR promotes Akt activation, and Akt promotes EGFR signalling in return, forming a positive feedback circle within the EGFR-Akt axis [52]. 

Another mechanism by which cells may resist apoptosis is related to the overexpression of the inhibitor of apoptosis protein (IAP) family of proteins [55]. Xiap directly inhibits the apoptotic activity of caspases, including caspase-3 and caspase-7 through its BIR2 domain and caspase-9 through its BIR3 domain [55,56]. During apoptosis, cells prevent the binding of XIAP to caspases and trigger its redistribution from the cytosol to the nucleus using endogenous antagonists of XIAP, such as second mitochondria-derived activator of caspases (SMAC), high-temperature-requirement A2 (HtrA2/Omi), endoplasmic reticulum aminopeptidase (ARTS), and XIAP associated factor 1 (XAF1) [55]. Furthermore, the internal ribosome entry segment (IRES) can initiate XIAP mRNA translation and enhance it using various IRES transacting factors, e.g., La autoantigen, heterogeneous nuclear ribonucleoproteins C1/C2 (hnRNP C1/C2) and MDM2 protein. Additionally, in cancer cells, the level of XIAP can be upregulated through phosphorylation by Akt kinase or by interaction with survivin, Notch receptor, or p34SEI-1 protein, which protects proteins by promoting degradation by ubiquitination [56]. In contrast, the upregulation in XIAP expression observed in cancer cells in response to DNA damage is associated with two proteins: Che-1 protein mediates activation of XIAP NF-κB-dependent transcription, while Mdm2 mediates XIAP by IRES-dependent translation [56]. In turn, XIAP overexpression provides resistance to apoptosis through the stimulation of both the intrinsic (mitochondrial directed) and extrinsic (death receptor directed) pathways [55].

The numerous genes involved in apoptosis indicate a highly complex interwoven network of checks and balances. In lung cancers, in addition to inhibition of pro-apoptotic proteins, chemotherapy resistance can be induced by activation or overexpression of anti-apoptotic molecules [57].

### 2.3. ABC Transporters

Overexpression of ATP-binding cassette (ABC) transporters plays an essential role in developing multiple drug resistance in NSCLC. ABC proteins have the ability to efflux a variety of small molecules, including toxic chemicals, from the cytosol by using energy from ATP hydrolysis [58,59]. The ABC protein family consists of 49 membrane proteins divided into seven subfamilies (ABCA—ABCG) expressed in various tissues [24,58,59,60]. 

Members of three subfamilies, viz. ABCB, ABCC, and ABCG (comprising at least 11 ABC superfamily transporters) are involved in the active efflux of anticancer drugs from the cytoplasm [24,60]. Thus, their overexpression can confer resistance to drugs such as cisplatin by lowering the intracellular accumulation of chemotherapeutics [61]. Exposure to one drug often elicits resistance to various structurally unrelated others. This phenomenon is related to the broad substrate specificity of ABC transporters [62]. In lung cancer cells, several ABC proteins are involved in the reduction of intracellular drug concentrations: ABCA1, ABCA2 (ABC transports not classified as multidrug-resistant proteins), ABCB1 (P-glycoprotein/multidrug resistance protein 1; MDR1), and the multidrug resistance-associated proteins (MRPs) ABCB4, ABCB11, and ABCC1-6 as well as ABCC10, ABCC11, and ABCG2 (BCRP/MXR) [58]. However, only ABCA1, ABCC2, and ABCC6 enable cisplatin resistance by direct efflux from the cell [63,64].

ABC transporters are integral membrane proteins typically consisting of evolutionarily conserved structures named nucleotide-binding domains (NBDs), which transfer the energy to transport the substrate across the membrane, and six α-helical transmembrane domains (TMDs), which provide the specificity for the substrate [24,60]. 

The NBD domains are typically located in the cytoplasm; they comprise 200–220 aa with an α-helical domain and a catalytic core domain. The latter includes most of the conserved regions, organised within the Walker A motif (or phosphate-binding P-loop) and Walker B motif (for the binding and hydrolysis of ATP) as well as the LSGGQ signature motif (involved in the binding of the nucleotide) and the A, D, H, and Q loops. One ATP molecule can be bound and hydrolysed by the Walker A and Walker B motifs of one NBD subunit and the C-loop and D-loop of the second subunit [24,60]. This ATP hydrolysis indicates conformational changes in the TMD domain, leading to alternating access from inside and outside of the cell, resulting in unidirectional transport across the cell membrane; it is also likely that ATP binding is sufficient to trigger NBD dimerisation and the transport of substrates [24]. While NBDs present an open conformation and are separated from one another in the absence of nucleotides, in the presence of ATP, they form a complete interface by approaching each other and “sandwiching” any bound ATP molecules [24,60]. ATP hydrolysis disrupts the dimer interface and releases the ADP and inorganic phosphate.

Additionally, effective coupling of substrates transport, utilised by binding ATP molecules, requires the transmission of the molecular motion from the NBD to the TMD domains. The interaction between TMDs and NBDs takes place on a coupling helix located in the cytoplasmic loops of the TMD [60]. Furthermore, several ABCC family transporters (ABCC1, -2, -3, -4, and -8) use GSH to enable the transport of several substrates. GSH conjugates present a higher affinity to transporters or act as stimulators of active transport [24]. In addition, the WNT/β-catenin pathway is an important signal transduction pathway that regulates tumour cell cisplatin resistance [63]. Activation of the WNT signalling pathway draws non-phosphorylated (activated) β-catenin into the nucleus, thus promoting the expression of downstream signalling molecules, including ABCB1, ABCC1, and ABCG2 and promoting the occurrence of cisplatin resistance in NSCLC [63,65].

### 2.4. Epigenetic Regulation by miRNAs

Cisplatin resistance is also regulated by miRNA, small endogenous non-coding RNA molecules consisting of about 18–23 nucleotides that influence posttranscriptional regulation of gene expression [27]. Their expression and wide range of targeted genes influence almost every genetic pathway from cell cycle checkpoint and cell proliferation to apoptosis. Although miRNAs expression correlates with various cancers, they may act as tumour suppressors and oncogenes depending on cancer type [66]. Furthermore, one particular miRNA acting as a tumour suppressor for one type of cancer may act as an oncogene in another histological subtype, i.e., oesophageal adenocarcinoma vs. squamous cell carcinoma [67]. 

One such miRNA is miRNA-630, which inhibits tumour growth and metastasis in oesophageal squamous cell carcinoma, hepatocellular carcinoma, and breast cancer, whereas it plays an oncogenic role in renal cell carcinoma, colorectal cancer, and gastric cancer [68]. Nevertheless, its role in cisplatin resistance of NSCLC remains unclear. MiRNA-630 targets and inhibits activation of p53, the master regulator of cisplatin-induced cell death, and blocks the early DNA damage response in lung cancer cells. It also reduces pro-apoptotic pathways regulated by p53 and targets distinct several other apoptotic modulators such as PARP3, DDIT4, EP300, and EP300 downstream effector p53, thus shifting the apoptotic balance towards cell survival [27,69]. Conversely, miRNA-630 inhibits cell proliferation by targeting cell-cycle kinase 7 (CDC7) [69]. In NSCLC cell models, miRNA-630 may confer cisplatin resistance in A549 cells while playing an opposite role in other lung adenocarcinoma cell lines: CL1-0 and H35869 [70,71]. This Janus face mechanism of action may be attributed to the fact that cancer cells usually mutate the TP53 gene favouring their survival and propagation. Certain mutant p53 proteins lose the wild-type activity and acquire oncogenic function, namely “gain-of-function”, to promote cancer development [53]. TP53 mutations are widespread in stages I through III of NSCLC [72]. A total of 34% of NSCLC patients have a mutation in the TP53 gene as an aftermath of frequent smoking [52]. Additionally, expression of p53 protein and its pro-apoptotic activity (in response to cisplatin treatment) in NSCLC was shown to be upregulated and enhanced after inhibition of miRNA-98-5p, thus proving this miRNA involvement in cisplatin resistance [73].

The most upregulated miRNA found in the cisplatin-resistant variant of NSCLC cell line A549, compared to parental A549, is miRNA-224 [74]. It targets potent cyclin-dependent kinase inhibitor p21WAF1/CIP, which is critical for p53, inducing cell cycle arrest, dysregulating G1/S cell cycle transition, and apoptosis, thus promoting tolerance to cisplatin [69,74].

Furthermore, one of the first discovered miRNA, miRNA-196a, which is upregulated in the vast majority of cancer types including NSCLC, was shown to be involved in the mediation of cisplatin resistance; however, its mechanism is not clear [75]. MiRNA-196a targets the Annexin-A1 (ANXA1) gene that regulates physiological mechanisms such as hormone secretion, apoptosis, exocytosis, and signal transduction [76]. ANXA1 is also involved in the acquisition and maintenance of a cancer stem cell-like phenotype that is characterised by upregulation of several chemoresistant mechanisms, including the activity of ABC proteins [77,78]. Furthermore, downregulation and silencing of microRNA-196a enhances the sensitivity of NSCLC cells to cisplatin treatment [75]. Moreover, miRNA-196a targets the 3′-UTR region of the HOXA5 gene that encodes the transcription factor homeobox protein (Hox-A5), resulting in increased NSCLC cell proliferation and metastasis [79].

Genes regulating the epithelial-mesenchymal transition (EMT) are the target of different miRNAs. MiRNA-15b upregulation is related to EMT, and its high expression was previously linked to the formation of nodules of metastatic lung cancers. MiRNA-15b targeting PEBP4 induces cisplatin resistance and is linked to overall poor prognosis [80]. One of the most important tumour suppressors in lung cancer is phosphatase and tensin homolog deleted in chromosome 10 (PTEN), which inhibits NSCLC cell growth by promoting G0/G1 arrest and cell apoptosis [81,82]. In many types of cancer (including NSCLC) aggressive phenotype correlates with downregulation of PTEN [83,84]. Until now, many miRNA were identified as targeting PTEN expression in human cancers let-7b, miRNA-106a, miRNA-142, miRNA-143, miRNA-21, miRNA-338, miRNA-340, miRNA-497, miRNA-503, and miRNA-582 [67,84,85,86]. Among them, namely miRNA-21, miRNA-92b and miRNA-328 confer cisplatin resistance in NSCLC [84,85,86].

Another target for miRNAs in cancer cells are the proteins involved in apoptosis. Here also we can observe both the oncogenic and suppressor activity of particular miRNAs. Two groups of oncogenic miRNAs: one activated by MET protooncogene (miRNA-103, miRNA-203) and the second by MET and EGF (namely miRNA-221, miRNA-222 and miRNA-30b/c) target the pro-apoptotic proteins such as APAF-1, BCL2-like11, PKC-ε or SRC [87]. As an effect, this can lead to skip the apoptotic signals, enhance cell survival, and finally develop the tumour necrosis factor (TNF) related apoptosis-inducing ligand (TRAIL) resistance in lung cancer cells [87,88].

#### MiRNAs Reducing the Cisplatin Resistance or Restoring the Sensitivity to Chemotherapeutics

However, certain miRNA enhance cisplatin sensitivity or reduce cisplatin resistance by targeting: ABCC2 that mediates cisplatin efflux or anti-apoptotic Bcl-xl by let7c, TGFβR2 by miRNA 17 family or MET by miRNA-206, in turn, inhibiting or reversing EMT phenotype, thus they are usually substantially downregulated in NSCLC [27,89,90,91].

Conversely, certain miRNAs exhibit tumour suppressor activity by increasing chemotherapeutics sensitivity or reducing the cisplatin resistance. For example, the let-7b mediated downregulation of anti-apoptotic Bcl-xl and ABCC2 leads to the cisplatin efflux decrease or the inhibition or reversing of EMT phenotype [27,89]. A similar effect will be mediated by the miRNA-17 family targeting TGFβR2, BECN1 or indirectly increasing the TIMP3 expression, which diminishes the ECM remodelling [90,92,93]. The decrease of miRNA-17-5p can cause paclitaxel resistance via BECN1 protein. However, inducing miRNA-17-5p overexpression in lung cancer cell lines leads to enhancing the cells’ sensitivity to paclitaxel [92]. Similarly, upregulation of other let7c, miRNA-130, miRNA-200, and miRNA-206 leads to sensitivity or reduction of cisplatin resistance [88,89,91,92,94]. The activity of MET-targeting miRNAs can reduce the resistance to cisplatin (miRNA-206) and TRAIL (miRNA-130a), thus exhibiting an effect opposite to protooncogenic miRNA-221 and miRNA-222 [88,91].

The most enigmatic miRNA involved in cisplatin resistance in NSCLC is miRNA-31, which targets the 3′-UTR region of the DICER1 gene [95]. Helicase with RNase motif, better known as Dicer, is a critical regulator of the biogenesis of miRNA and small interfering RNA (siRNA) [96]. Thus, downregulation of Dicer by miRNA-31 leads to overall downregulation of miRNA production, both oncogenic (involved in the acquisition of cisplatin resistance) and tumour suppressor (that renders NSCLC sensitisation to cisplatin treatment) [95].

Recent data suggests that acquired chemoresistance may be transferred to sensitive cells by extracellular vesicle as their cargo contains multiple particles, including proteins, mRNA and miRNA [97]. Exosomes present in tumour microenvironments can be internalised by adjacent cells and modify the phenotype of the recipient cell to reflect the regulatory functions of the exosome cargo. This phenomenon may be observed within the same tumour or at other anatomical sites [98]. Our recent research proved that exosome-derived miRNA poses diagnostic value in early NSCLC diagnosis; however, possible prognostic values for cisplatin-based therapy outcome based on the miRNA panel are not yet determined [2]. Representative miRNAs involved in cisplatin resistance are presented in Table 1.

### 2.5. Cisplatin Resistance and the Tumour Microenvironment (TME)

**Figure 2:**
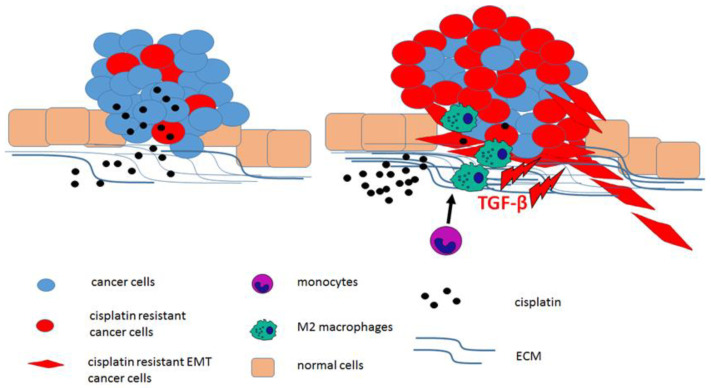
TME impact on cisplatin resistance. Cisplatin administration leads to the selection and acquisition of cisplatin-resistant cancer cells. The TME restricts the diffusion rate of cisplatin. Cisplatin-resistant cancer cells support the activation of M2 macrophages, which in turn induce EMT via TGF-β secretion, resulting in enhanced chemoresistance and metastatic potential.

The tumour microenvironment (TME) consists of both normal, non-malignant tissue cells and immune cells with diverse phenotypes and functions that can strongly modulate the response to chemotherapy and increase metastatic potential [101]. The least complicated TME activity leading to cisplatin resistance is the formation of a physiological barrier composed of a dense extracellular matrix (ECM) and closely packed cells around the tumour, which substantially restricts the diffusion rate of anticancer drugs into cancer cells [102]. The region comprising the tumour and the TME is often named the “wound that does not heal”, as both states are characterised by similar molecular mechanisms, including inflammation [24]. One of the key components of the TME is tumour-associated macrophages (TAMs), which are responsible for promoting EMT, migration, tissue infiltration, dissemination, and thus distant metastasis [103]. Generally, monocytes undergo differentiation towards one of two subpopulations: M1 (classical) or M2 (alternative) macrophages. Alternative activation leads to the formation of regulatory macrophages and wound-healing macrophages. The activation of the M2 form results in the release of TGF-β, thus triggering EMT and increasing the metastatic potential of cancer cells [104]. EMT is considered to be a significant factor in chemoresistance, converting stationary epithelial cells into mobile, less proliferative mesenchymal cells [105]. In NSCLC, TAMs increase the population of CD133+ expressing cancer stem cells (CSCs); they also enhance the expression of genes associated with the inflammation proteins Sox2 and NF-κB [101]. Furthermore, cisplatin-resistant NSCLC cells present elevated expression of other oncogenic and stemness markers, such as Src, Notch1, macrophage inhibitory factor (MIF), and CD155, which promote alternative activation of TAMs into pro-tumourigenic M2 (-like) macrophages [103]. Furthermore, cisplatin-stimulated classically activated macrophages (CAMs) enhance ovarian cancer cell migration, triggering EMT via the CCL20/CCR6 axis [106]. The CCL20/CCR6 axis promotes NSCLC disease progression, and high expression of CCR6 has been associated with shorter disease-free survival [107]. The relationships between cisplatin resistance and the TME are summarised in Figure 2.

Other factors that dictate NSCLC cells behaviour are different immune checkpoints markers, including programmed cell death ligand 1 (PD-L1) [108]. PD-L1, a 40-kDa transmembrane protein, the major ligand for programmed cell death (PD-1), is a cell surface protein in the B7 family that modulates the immune response through the inhibition of T-cell function and proliferation, including cell apoptosis, and creates cancer resistance [108,109,110]. 

The increased PD-L1 expression level on tumour cells was found to be associated with poor prognosis and cancer aggressiveness [111]. Recent studies have shown that cisplatin-resistant NSCLC cells present higher PD-L1 expression [109] while PD-L1 silencing enables overcoming cisplatin resistance. miRNA-200 and ZEB1 axis, which are known to control migration and invasion and EMT, can also regulate PD-L1 expression. A decrease in PD-L1 expressions was reported due to ectopic miRNA-200 expression or ZEB1 knockdown models. Low miRNA-200 with high ZEB1 and PD-L1 expressions in mesenchymal tumours created a microenvironment of decreased CD8+ T-cells populations [108]. Moreover, resistant cells undergo an epithelial-mesenchymal transition to enable invasion and metastasis and escape immune surveillance by expressing PD-L1/PD-1 [108]. Another microRNA targeting the PD-L1 expression is miRNA-197. Silencing of the miRNA-197 increases the PD-L1 expression and may be the parallel method leading to the adherence of chemoresistance [112]. 

The PD-1 and its ligand focus on the modulation of anti-PDL-1 therapies, leading to inhibition of the PD-1/PD-L1 axis [113]. The immunotherapy with PD-1 or its ligand can improve the survival rate in NSCLC patients. Assessing the protein level by itself may be used as a predictive factor for using the immune checkpoint inhibitors [114]. Conversely, only a small subset of NSCLC patients will benefit from inhibition PD-1/PD-L1 axis because the cancer cells will acquire drug resistance, leading to progression of the disease [115]. 

Additionally, the cancer microenvironment and cancer mass itself substantially differ from normal, healthy tissue. The metabolic alterations of cancer cells that distinguish them from healthy cells are recognized as one of the ten hallmarks of cancer. An altered metabolism helps cancer cells to sustain high proliferative rates in a hostile environment resulting from poor vascularization, which limits the supply of oxygen [116]. In the 1920s, Otto Warburg postulated that tumour cells consume glucose and excrete lactate at a significantly higher rate compared to healthy resting cells [117]. This phenomenon is currently named the “Warburg effect”. Warburg effect has been proposed to be an adaptation mechanism to support the biosynthetic requirements of uncontrolled proliferation. Cancer cells utilise glycolysis in normoxia conditions as primary glucose metabolism. Per unit of glucose, aerobic glycolysis is an inefficient means of generating ATP compared to the amount obtained by mitochondrial respiration (oxidative phosphorylation; OXPHOS). However, the rate of glucose metabolism through aerobic glycolysis is higher and the amount of ATP synthesized are comparable when either form of glucose metabolism is utilized. Another proposed mechanism to account for the biosynthetic function of the Warburg Effect is the regeneration of NAD+ from NADH in the pyruvate to lactate step that completes aerobic glycolysis. In this scenario, NADH that is produced by GAPDH must be consumed to regenerate NAD+ to keep glycolysis active. This high rate of glycolysis allows for supply lines to remain open that can, for example, siphon 3-phosphoglycerate (3PG) to serine for one-carbon metabolism-mediated production of NADPH and nucleotides [118]. Importantly, utilization of glycolysis by cancer cells decreases the number of generated ROS, leading to increased resistance to DNA damaging chemotherapeutics such as cisplatin. Furthermore, cells relying on glycolysis are less likely to undergo apoptosis as mitochondrial membrane permeabilization by Bax and Bak, which is a critical step in its induction, and OXPHOS is known to activate Bax and Bak [119]. In prostate and pancreatic cancer it has been shown that exosomes promote glycolysis and block oxidative metabolism, as they may deliver microRNAs that silence oxidative metabolism genes [120].

## 3. Protein-Protein Interaction Changes in NSCLC Caused by the Acquisition of Cisplatin Resistance

**Figure 3:**
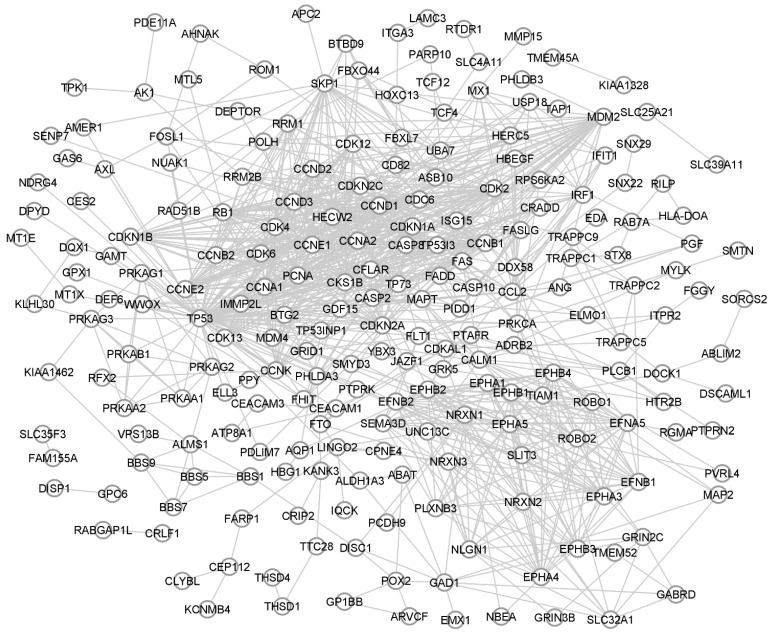
PPI network of 250 top differently expressed genes between cisplatin resistant vs. parental A549 cells. The PPI pairs were imported and visualised by Cytoscape software.

Resistance to cisplatin is acquired through many ostensibly unrelated mechanisms. To demonstrate this multifactorial nature, several studies have analysed changes in mRNA expression caused by cisplatin resistance in an NSCLC cellular model using the Gene Expression Omnibus database [121] GSE108214, listing the mRNA expression profiles of the parental and cisplatin-resistant NSCLC cancer cell line A549. The findings were processed using the GEO2R online analytical tool [122]. It was found that over 29,000 genes were differently expressed between resistant and sensitive A549 cells. A protein-protein interaction (PPI) network was created using the top 250 differently expressed genes (DEGs) and using STRING version 11.0 online software [123] and the Cytoscape open-source software platform for visualising complex networks [124]. KEGG (Kyoto Encyclopedia of Genes and Genomes) pathways analysis was performed using DAVID online tool [125] and KEGG PATHWAY database [126]. The PPI network, composed of 250 top differently expressed mRNAs enriched by known signalling proteins, is shown in Figure 3.

The PPI network indicated the most efficiently networking DEGs that are strongly related to cisplatin resistance. Among them, three of the top five (TP53, MDM2, and CDKN1A; Table 2) are reviewed in the apoptosis section as important anti-apoptotic factors involved in repairing platinum-derived DNA damage. Furthermore, the next two, proliferating cell nuclear antigen (PCNA) and DNA polymerase eta (POLH)—a member of the Y-family of DNA polymerases, mediates DNA translesion synthesis, and are thus involved in the primary mechanism for DNA damage tolerance (as reviewed in the post-replication repair section). An analysis of the most dysregulated KEGG pathways (Table 3) demonstrated the complexity of cisplatin resistance mechanisms and highlighted how mutually complementary they are. The KEGG pathways hsa01524, i.e., platinum drug resistance and hsa04210 and apoptosis, are composed of several sub-pathways, including PI3K-Akt and p53; these are also dysregulated, resulting in increased cell survivability. The observed dysregulation of focal adhesion and tight junctions may also suggest phenotypical changes towards a more aggressive and motile mesenchymal phenotype following EMT.

## 4. Conclusions

Cisplatin resistance occurs due to multiple complex mechanisms operating at different cellular levels that either inhibits apoptosis, promotes cell survival, or acts simultaneously. Resistance to cisplatin is a significant impediment in NSCLC chemotherapy. Resistance can be enhanced by reducing cellular cisplatin levels, increasing inactivation by endogenous nucleophiles, altering the expression of regulatory genes, increasing repair of adducts, and increasing adduct tolerance. An improved understanding of cisplatin resistance will better identify therapeutic targets and allow a more accurate prediction of clinical response. Additionally, it will allow therapy to be better tailored to the needs of individual patients.

## Figures and Tables

**Table 1:** MiRNAs involved in drug resistance regulation in NSCLC.

miRNA	Target Gene(s)	Chemoresistance	References
↑miRNA-15b	↓PEBP4	TKIs resistance	[80]
↑miRNA-20	↓MMP2	paclitaxel resistance	[93]
↑miRNA-21,	↓PTEN, BCL2, IGF1R	TKIs resistance	[67]
↑miRNA-21,miRNA-30c, ↑miRNA-100	↓caspase-3, caspase-8, TRAF7, FoxO3a ↑NF-κB signalling	TRAIL resistance	[99]
↑miRNA-21, miRNA-92b, miRNA-328	↓PTEN	cisplatin resistance	[84,85,86]
↑miRNA-31,	↓DICER1, ABCB9	cisplatin resistance	[96]
miRNA-98-5p	↓MAPK signalling	cisplatin resistance EGCG-cisplatin resistance	[73]
↑miRNA-30b/c, miRNA-221/222	↓APAF-1, BIM ↓SRC, PKC-ε	TKIs resistance	[100]
↑miRNA-196a	↓Annexin-A1, HOXA5	cisplatin resistance	[75,79]
↑miRNA-221/222	↓PTEN, TIMP3	TRAIL resistance	[87,88]
↑miRNA-224	↓p21WAF1/CIP	cisplatin resistance	[69,74]
↑miRNA-630	↓CDC7	cisplatin resistance	[27,69]
↑Let-7b	↓ABCC2, BCL-XL	↑sensitivity to cisplatin	[89]
↑miRNA-17-5p	↓BECN1, TGFβR2	↑sensitivity to cisplatin ↑sensitivity to paclitaxel	[92]
↑miRNA-17-5p	↑TIMP	reversing of EMT phenotype, ECM remodelling	[93]
↑miRNA-130	↓MET, miRNA-221, miRNA-222.	↑sensitivity to TRAIL	[88]
↑miRNA-200, miRNA-206	↓p70S6K1, ↓HIF-1α	↑sensitivity to cisplatin	[94]

↑: An increased level of given expression of miRNA/gene leading to the formation of chemoresistance/observed phenomena/mechanisms. ↓: The decreased level of given expression of miRNA/gene leading to the formation of cisplatin resistance/observed phenomena/mechanisms.

**Table 2:** Top 5 networking DEGs involved in cisplatin resistance—upregulated in cisplatin-resistant A549 cells. Fold change—A549 cisplatin-resistant vs. A549 parental, degree—presents the number of undirected edges.

Gene	Betweenness	Degree	Adj. *p* Value	*p* Value	Fold Change
*TP53*	0.499325647	86	0.52935846	2.04 × 10^−1^	1.3603568
*MDM2*	0.04074928	41	0.00026943	1.32 × 10^−6^	10.4840794
*CDKN1A*	0.02606798	40	0.00032013	1.83 × 10^−6^	10.1108217
*PCNA*	0.005309797	24	0.02632428	1.48 × 10^−3^	4.3818849
*POLH*	0.0002545446	5	0.00054359	4.25 × 10^−6^	9.1942649

**Table 3:** Major dysregulated KEEG pathways. FDR—falls detection rate < 0.05; *p* value of < 0.05.

KEGG	Pathway Name	Count in Network	FDR
hsa01524	Platinum drug resistance	9 of 70	2.58 × 10^−5^
hsa04210	Apoptosis	27 of 135	7.38 × 10^−23^
hsa04310	Wnt signalling pathway	11 of 143	0.00016
hsa04115	p53 signalling pathway	22 of 68	9.40 × 10^−19^
hsa04151	PI3K-Akt signalling pathway	21 of 348	2.28 × 10^−6^
hsa04510	Focal adhesion	10 of 197	0.0049
hsa04530	Tight junction	11 of 167	0.00048

## Data Availability

Gene Expression Omnibus data set #GSE108214: https://www.ncbi.nlm.nih.gov/geo/query/acc.cgi?acc=GSE108214.
